# Trends in healthcare utilization and costs associated with pneumonia in the United States during 2008–2014

**DOI:** 10.1186/s12913-018-3529-4

**Published:** 2018-09-14

**Authors:** Sabine Tong, Caroline Amand, Alexia Kieffer, Moe H. Kyaw

**Affiliations:** 1 IVIDATA Stats, 79 Rue Baudin, 92300 Levallois-Perret, France; 2 Sanofi, 1 Avenue Pierre Brossolette, 91380 Chilly-Mazarin, France; 3 Sanofi Pasteur, 14 Espace Henry Vallée, 69007 Lyon, France; 4 Sanofi Pasteur, Swiftwater, 1 Discovery Drive, Swiftwater, PA 18370 USA; 5 Present address: Boehringer Ingelheim, 900 Ridgebury Road, Ridgefield, CT 06877 USA

**Keywords:** Pneumonia, Healthcare utilization, Healthcare cost, United States, Epidemiology

## Abstract

**Background:**

Pneumonia is the leading cause of morbidity and mortality worldwide. Pneumococcal conjugate vaccines have reduced the burden of pneumonia, but data on the current burden of pneumonia and its impact on the healthcare system are needed to inform the development and use of new vaccines and other preventive measures.

**Methods:**

We retrospectively analyzed the frequency of pneumonia in the US during 2008–2014 using data from the MarketScan® Commercial Claims and Encounters database. Frequencies of healthcare utilization related to the index pneumonia episode were calculated using the annual number of enrolled person-years (PY) as the denominator and the number of individuals with pneumonia as the numerator. Pneumonia-associated costs were calculated as mean payment per episode during the 2 years from 2013 to 2014.

**Results:**

The overall annual healthcare utilization rate for pneumonia was 15.1 per 1000 PY and decreased slightly from 2008 to 2014 (from 15.4 to 13.5 per 1000 PY). Most pneumonia-related healthcare utilization was due to office/outpatient visits (10.3 per 1000 PY; 68.3%). Emergency department/urgent care visits (2.5 per 1000 PY; 16.9%) and hospitalizations (2.2 per 1000 PY; 14.8%) contributed less. Pneumonia-related healthcare utilization was highest in children < 5 years (rate per 1000 PY = 29.7 for < 1 year, 47.9 for 1 year, and 39.5 for 2–4 years) and adults > 65 years (45.0 per 1000 PY). The mean cost per pneumonia episode (95% confidence interval) was US$429.1 ($424.8–$433.4) for office/outpatient visits, $1126.9 ($1119.5–$1134.3) for emergency department/urgent care visits, and $10,962.5 ($10,822.8–$11,102.2) for hospitalization.

**Conclusions:**

The burden of pneumonia on the US healthcare system remains substantial. The results presented here can help guide new vaccination strategies and other preventive interventions for reducing the remaining burden of pneumonia.

**Electronic supplementary material:**

The online version of this article (10.1186/s12913-018-3529-4) contains supplementary material, which is available to authorized users.

## Background

Pneumonia is the leading cause of death due to infection worldwide in children aged < 5 years and is responsible for approximately 16% of the 5.6 million deaths in this population [1, 2]. Pneumonia is also an increasing problem for older adults, who are at the highest risk for pneumonia-related hospitalization and death [3–5]. Pneumonia is mostly caused by the 97 known serotypes of *Streptococcus pneumoniae* [6], although it can also be caused by viruses, fungi, other bacteria, and other microorganisms [7]. Besides low or high age, other main risk factors include smoking, chronic disease, a weakened immune system, and being hospitalized [8]. In the US, the direct annual cost of community-acquired pneumonia has been estimated to be at least $17 billion [9], and in Europe, overall annual costs are estimated to be € 10.1 billion [10].

A 23-valent pneumococcal polysaccharide vaccine was introduced in the US in 1983 [11], followed a 7-valent pneumococcal vaccine (PCV7) in 2000 [12] and a 13-valent pneumococcal vaccine (PCV13) in 2010 [13]. Although the impact of the 23-valent pneumococcal polysaccharide vaccine is uncertain [14], PCVs substantially decreased the incidence of pneumonia [15–17]; however, PCVs protect only against the 7 or 13 serotypes included and therefore offer limited protection against all-cause pneumonia [7, 16]. Recommendations in the US are for vaccination of all children aged < 2 years with a PCV; of all adults aged ≥65 years and persons aged 2–64 years with certain medical conditions with PPV23 or PCV; and of adults aged 19–64 years who smoke cigarettes with PPV23 [18].

To develop and implement new pneumococcal vaccines and other preventive measures, further data on the current burden of pneumonia and its impact on the healthcare system are needed. In this study, we examined US insurance records to determine the annual frequency of pneumonia during 2008–2014 and the costs associated with index pneumonia events during the two most recent years (2013–2014).

## Methods

### Study design

This was a retrospective analysis of pneumonia-related healthcare utilization in the US. The analysis used data extracted from the Truven Health MarketScan® Commercial Claims and Encounters database [19]. The co-primary objectives were (a) to determine the annual and monthly frequency of pneumonia-related healthcare utilization from 2008 to 2014 and (b) to determine pneumonia-associated costs in the two most recent years (2013–2014) due to hospitalization, outpatient visits, and emergency department (ED)/urgent care (UC) visits.

### Data source and extraction

The MarketScan database contains information on US individuals insured commercially (i.e. privately) or through the Medicare program [19]. The database collects information on paid claims from health plans, employers, and state-level Medicaid agencies using a nationwide convenience sample. It covers all census regions of the US, includes an average of 48,982,662 individuals per year, and has complete longitudinal records of patient demographics, outpatient services, inpatient services, long-term care, and prescription drug claims. The database is considered representative of the US population with employer-provided health insurance [1, 20] and is used extensively to understand the burden and healthcare utilization for different illnesses in the country. Records within the database are de-identified and fully compliant with US patient confidentiality requirements, including the Health Insurance Portability and Accountability Act of 1996. For this reason, ethical approval was not required for this study.

In this study, data were extracted from January 1, 2008 to December 31, 2014. Only data from individuals in the enrollment tables were included. Pneumonia episodes were identified as a consultation with a principal diagnosis of pneumonia or with a principal diagnosis of meningitis, septicemia, or empyema in addition to a diagnosis of pneumonia in another diagnostic field [21]. For outpatient visits, the principal diagnosis was considered based on International Classification of Diseases, 9th revision, Clinical Modification codes in the primary or the secondary position (see Additional file 1: Table S1). In addition, the first consultation had to be more than 28 days after any previous consultation that had the same diagnosis code. Index episodes were defined as the first episode of pneumonia occurring in the calendar year. Extracted data included the total enrollment numbers for each year; demographic data, including age, sex, geographic region (Northeast, North Central, South, West, or unknown), and insurance type (commercial, Medicare); and the amounts for adjudicated claims paid by health plans, insurers, and patients.

### Outcome definitions and measures

Outcome measures included the number of pneumonia cases (overall and by geographical region and insurance type); index visit demographics (mean and median age, age range, and sex distribution); proportions of pneumonia cases for each setting (hospitalization, outpatient visits, and ED/UC visits) for all ages and each age group; annual frequency of pneumonia-related healthcare utilization overall and by setting of the index visit for all ages and each age group; and monthly frequency of pneumonia. For patients transferred to several services within the same day, the setting was defined as the most severe (i.e. hospitalization > ED/UC > outpatient). Pneumonia-associated costs during 2013 and 2014 were determined overall and by setting for the index visit and all follow-up visits that had the same diagnosis code occurring within 28 days. Costs were based on paid amounts of adjudicated claims, including health plan and insurer payments and patient cost-sharing (i.e., copayments, deductibles, and coinsurance). Total costs were estimated as the sum of all costs in each individual setting.

### Statistical analysis

The annual frequency of pneumonia is given per 1000 person-years (PY) and was calculated as 1000 × [annual number of patients with a pneumonia episode] ÷ [annual number of total enrolled PY in the MarketScan databases]. Monthly frequencies for each year were calculated per 1000 person-months. Proportions were calculated as 100% × [number of index visits for each setting] ÷ [number of total index visits]. Costs related to the index episode were calculated as the mean payment for each episode during the 2 years from 2013 to 2014. The 95% confidence intervals (CIs) for frequencies of pneumonia and costs were calculated using a normal approximation.

The significance in the difference in frequencies of pneumonia between 2008 and 2009 and 2014 was assessed by a log-linked Poisson regression with the log of the number of PYs as an offset. The exposure variable was the number of patients with pneumonia for a given year and age group. Year, age group, and interaction between year and age group were included as predictors in the model. A *p*-value < 0.05 was considered to indicate a significant difference.

Analyses were performed using SAS® Enterprise Guide 7.1 (SAS Institute, Cary, NC, USA).

## Results

### Pneumonia cases

Between January 1, 2008 and December 31, 2014, an average of 41,610,536 PY were included in the database each year. On average, 626,580 pneumonia cases were diagnosed each year.

### Demographics of pneumonia cases

Slightly less than half of pneumonia patients were male (48.7% overall) and the mean age was 41.9 years (Table 1). Overall, 31.1% of pneumonia cases were among children and adolescents (< 18 years), 44.8% were among non-elderly adults (18–64 years), and 24.1% were among elderly adults (≥ 65 years). In all years, over 71% of patients with pneumonia were commercially insured (Additional file 2: Table S2). Most pneumonia cases were reported in the South (36.8% overall) and North Central region (26.4% overall) (Table 1), although the proportion decreased in both regions from 2008 to 2014 (Additional file 2: Table S2).

**Table 1:** Demographic characteristics of pneumonia patients in the MarketScan database between 2018 and 2014

Variable	Mean	Range of annual values
Pneumonia patients, n	626,580	486,442–758,196
Age (y)		
Mean	41.9	39.4–44.7
Median	46	42–50
Age range, %		
< 1 y	2.0	1.6–2.4
1 y	3.3	2.9–3.6
2–4 y	8.7	7.7–9.3
5–17 y	17.1	15.1–19.3
18–49 y	22.8	21.4–24.2
50–64 y	22.0	20.9–23.8
65–74 y	8.3	7.0–10.1
75–84 y	9.2	8.3–10.3
≥ 85 y	6.7	5.4–8.1
Male, n (%)	48.7	48.4–48.8
Insurance, n (%)		
Commercial ^a^	75.5	71.1–79.0
Medicare ^b^	24.5	21.0–28.9
US geographic region, n (%)		
Northeast	17.5	9.7–22.6
North Central	26.4	24.1–29.8
South	36.8	31.2–45.4
West	17.7	14.5–21.2
Unknown	1.7	0.2–3.1

^a^ Includes active employees and their dependents, early (non-Medicare) retirees and their dependents, and individuals covered under the Consolidated Omnibus Budget Reconciliation Act

^b^ Includes retirees (> 65 y), their dependents, and younger people with disabilities

### Frequency of pneumonia cases

The mean annual frequency of pneumonia between 2008 and 2014 was 15.1 per 1000 PY (Table 2). Based on this and the US population in 2016 (322,762,018), an estimated 4.9 million patients suffer from pneumonia each year. The mean rate per 1000 PY was highest in children < 5 years of age (29.7 for < 1 year, 47.9 for 1 year, and 39.5 for 2–4 years) and elderly adults (45.0 for ≥65 years). In elderly adults, the frequency of pneumonia increased with age (mean rate per 1000 PY = 28.2 for 65–74 years, 53.4 for 75–84 years, and 94.2 for ≥85 years).

**Table 2:** Frequency of index pneumonia cases per 1000 person-years by setting and age group between 2008 and 2014

Age group	Frequency per 1000 person-years	
	Mean	Range of annual values
< 1 y	29.7	22.5–34.7
1 y	47.9	39.9–52.9
2–4 y	39.5	33.9–43.2
5–17 y	14.9	12.8–18.0
18–49 y	7.6	6.8–8.9
50–64 y	13.9	13.1–15.2
65–74 y	28.2	26.4–30.9
75–84 y	53.4	51.6–55.6
≥ 85 y	94.2	84.3–98.6
Overall	15.1	13.5–16.6

**Fig. 1:**
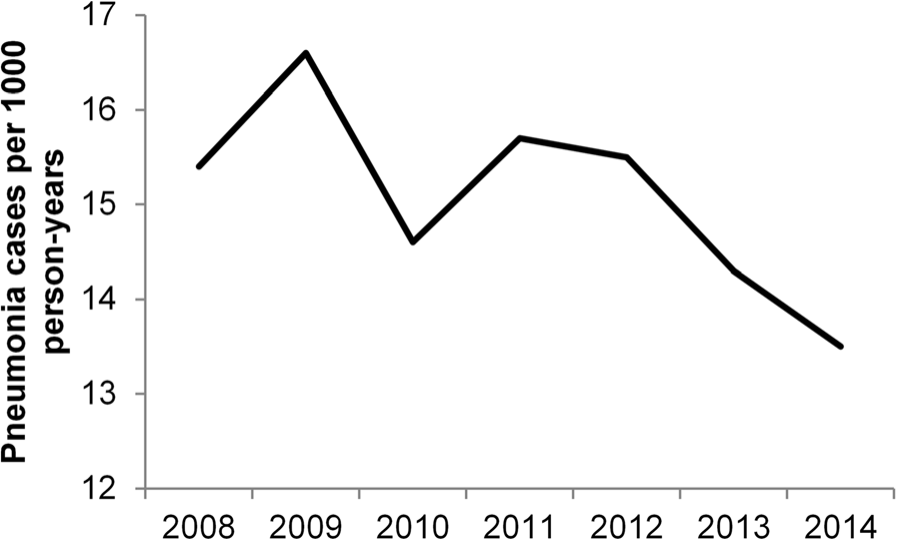
Overall rate of index pneumonia cases between 2008 and 2014

The overall mean frequency of pneumonia decreased by 16.1% from 16.1 per 1000 PY in the pre-PCV13 period (2008–2009) to 13.5 per 1000 PY in 2014 (*p* < 0.0001 for change over time) (Fig. 1 and Table 3). The overall rate decreased significantly for all age groups < 85 years of age and increased significantly for individuals ≥85 years (Table 3). The greatest decrease (− 34.8%) was for children < 1 year of age. The annual frequency of pneumonia cases per 1000 person-years by age groups during the period 2008-2014 are shown in Additional file 3: Table S3.

**Table 3:** Change in frequency of pneumonia cases per 1000 person-years from 2008 to 2009 to 2014

Age group	Frequency per 1000 person-years		Change (%)	*P*-value ^b^
	2008–2009 ^a^	2014		
< 1 y	34.6	22.5	−34.8	< 0.0001
1 y	51.4	39.9	−22.4	< 0.0001
2–4 y	40.9	33.9	−17.2	< 0.0001
5–17 y	15.9	12.8	−19.5	< 0.0001
18–49 y	8.6	6.8	−20.7	< 0.0001
50–64 y	15.0	13.1	−12.7	< 0.0001
65–74 y	29.4	26.4	−10.1	< 0.0001
75–84 y	53.4	52.6	−1.5	0.0054
≥ 85 y	89.1	95.7	+ 7.4	< 0.0001
Overall	16.1	13.5	−16.1	< 0.0001

^a^ Average for 2008 and 2009

^b^*P*-value for change over time determined by Poisson regression analysis

**Fig. 2:**
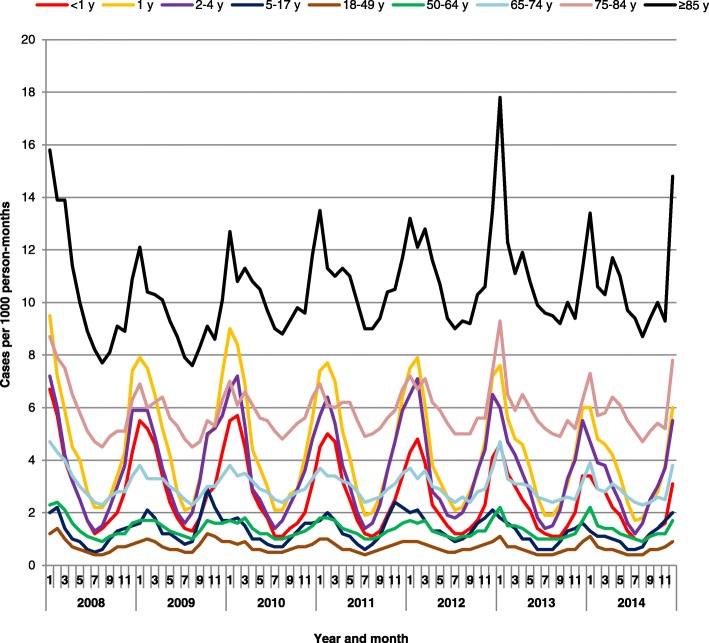
Seasonality of frequency of pneumonia cases per 1000 person-months in the US, 2008–2014

Seasonality of pneumonia was consistent during these years, with a peak in the winter months in all age groups (Fig. 2).

### Proportion of pneumonia cases by setting

Overall, between 2008 and 2014, most of the index pneumonia episodes (68.3%) were related to an outpatient visit (Table 4), followed by ED or UC visits (16.9%) and hospitalization (14.8%). This was also the case for each age group (Table 4) and each year (Additional file: 4 4: Table S4), although in elderly adults, pneumonia cases were diagnosed as frequently in inpatient (42.0–53.4%) as in outpatient settings (46.6–58.0%). The proportion of index events of pneumonia in the hospital setting appeared to be highest in elderly adults, with averages of 27.4% (range 26.2–28.3%) for 65–74 years of age, 35.2% (range 34.1–36.4%) for 75–84 years of age, and 38.6% (range 37.0–40.2%) for those aged ≥85 years.

**Table 4:** Proportion of pneumonia cases by setting and age group between 2008 and 2014

Age group	Setting	Proportion of pneumonia cases (%)	
		Mean	Range
< 1 y	Hospitalization	11.9	10.4–13.8
	ED/UC visits	25.3	23.0–26.9
	Outpatient visits	62.8	62.0–64.4
1 y	Hospitalization	6.8	5.4–8.3
	ED/UC visits	22.9	21.9–23.6
	Outpatient visits	70.3	69.5–71.3
2–4 y	Hospitalization	4.4	3.6–5.4
	ED/UC visits	15.8	15.1–16.6
	Outpatient visits	79.8	78.7–80.7
5–17 y	Hospitalization	3.2	2.6–3.9
	ED/UC visits	12.3	11.0–13.8
	Outpatient visits	84.6	83.5–85.1
18–49 y	Hospitalization	8.5	7.7–9.3
	ED/UC visits	22.2	18.3–27.9
	Outpatient visits	69.4	63.7–73.4
50–64 y	Hospitalization	15.6	14.7–16.3
	ED/UC visits	16.1	13.3–19.3
	Outpatient visits	68.3	65.0–71.5
65–74 y	Hospitalization	27.4	26.2–28.3
	ED/UC visits	14.6	12.8–15.9
	Outpatient visits	58.0	57.3–59.0
75–84 y	Hospitalization	35.2	34.1–36.4
	ED/UC visits	14.9	13.8–16.5
	Outpatient visits	49.9	49.0–51.0
≥ 85 y	Hospitalization	38.6	37.0–40.2
	ED/UC visits	14.8	13.7–16.2
	Outpatient visits	46.6	44.8–48.8
Overall	Hospitalization	14.8	13.5–15.7
	ED/UC visits	16.9	14.9–19.3
	Outpatient visits	68.3	65.8–70.2

Abbreviations: *ED* emergency department, *UC* urgent care

### Costs of index pneumonia episodes during 2013–2014

During the 2 years from 2013 to 2014, the total mean cost per pneumonia episode was $2783.1 (95% confidence interval [CI], 2754.4–2811.8) (Table 5). Outpatient visits accounted for most episodes of pneumonia (85.8%) and had a mean cost of $429.1 (95% CI, 424.8–433.4) per episode. ED/UC visits (29.5% of all episodes) had a mean cost of $1126.9 (95% CI, 1119.5–1134.3) per episode, and hospitalizations (19.2%) had a mean cost of $10,962.5 (95% CI, 10,822.8–11,102.2) per episode.

**Table 5:** Mean costs related to the index pneumonia episode overall and by setting between 2013 and 2014

Setting	Proportion of pneumonia episodes (%)^a^	Cost per episode (US$)	
		Mean	95% CI
Hospitalized	19.2	10,962.5	10,822.8–11,102.2
ED/UC	29.5	1126.9	1119.5–1134.3
Outpatient	85.8	429.1	424.8–433.4
Overall	100.0	2783.1	2754.4–2811.8

Abbreviations: *CI* confidence interval, *ED* emergency department, *UC* urgent care

^a^Patients may have received several services, so total may be > 100%

The cost of pneumonia ranged from $910.2 to $2621.9 for children, from $2177.7 to $3478.3 for adults, and from $4025.8 to $4993.0 for elderly adults (Table 6). The mean cost of pneumonia-related outpatient visits in elderly ≥85 years was 1.6 to 7 times higher than in other age groups. Also, hospitalizations related to the index episode of pneumonia were more than two times more frequent than in children < 1 year, although the mean cost associated with hospitalization was half as much as in children < 1 year.

**Table 6:** Mean costs related to the index pneumonia episode by age group between 2013 and 2014

Age group	Cost per episode (US$)	
	Mean	95% CIs
< 1 y	2621.9	2244.7–2999.1
1 y	1255	1192.0–1318.0
2–4 y	923	879.3–966.7
5–17 y	910.2	866.6–953.8
18–49 y	2177.7	2112.3–2243.1
50–64 y	3478.3	3412.4–3544.2
65–74 y	4025.8	3917.8–4133.8
75–84 y	4605.1	4499.6–4710.6
≥ 85 y	4993	4882.5–5103.5

Abbreviation: *CI* confidence interval

^a^Patients may have received several services, so total may be > 100%

Based on our estimate of 4.9 million pneumonia cases per year for the total US population, estimated annual costs would be US$ 13.4 billion.

## Discussion

Although recommendations in the US are for pneumococcal vaccination for all children aged < 2 years, adults aged ≥65 years, and persons aged 2–64 years with certain medical conditions [18], pneumonia-associated healthcare utilization and costs in the US remained substantial between 2008 and 2014. Based on our findings, we estimate that 4.9 million patients suffer from pneumonia annually in the US, resulting in US$ 13.4 billion in costs related to the index episode. This study also showed that children aged < 5 years and elderly adults, especially the oldest individuals, had the highest rates of pneumonia.

These findings are not surprising because PCVs, the most frequently used pneumococcal vaccines, cover only the 7 or 13 included serotypes and therefore offer limited protection against all-cause pneumonia [7, 16]. Other common causes of pneumonia include non-vaccine serotypes of *S. pneumoniae*, along with *Haemophilus influenzae*, *Staphylococcus aureus*, influenza virus, and pulmonary diseases [7]. Of the non-pneumococcal causes of pneumonia, vaccines are available only for influenza virus, for which uptake remains suboptimal [22].

Over the study period, the frequency of pneumonia decreased significantly, especially for children ≤1 year of age, suggesting an increasing impact of vaccination and other preventive measures. In contrast, the frequency of pneumonia for adults aged ≥85 years increased, which agrees with other studies indicating that pneumonia is an increasing problem for older adults [3–5]. Overall, most pneumonia cases, including those in young children, were seen in outpatient settings, although about half of the cases in elderly adults were seen in an inpatient setting, further highlighting the growing problem of pneumonia in older adults and the burden it places on the healthcare system.

We were unable to find published reports on changes in overall rates of pneumonia for the same period as the current study (2008–2014), although data from the National (Nationwide) Inpatient Sample indicated that the age-adjusted in incidence of hospitalized cases of pneumonia decreased in the US by roughly one-quarter between 2001 and 2014 [23]. Also, Tennessee hospital discharge data from 1998 to 2012 indicated that hospitalization for pneumonia in children aged < 2 years decreased by 72% from 1998 (pre-PCV period) and 2012 (2 years after PCV13 was introduced) and by 27% from 2000 to 2010 (PCV7 period) and 2012 [24]. Another study of hospital discharge data found a decrease of 17–21% in children aged < 5 years after PCV13 was introduced [25]. Data from the Medical Expenditure Panel Survey database indicated a 16% decrease in the incidence of pneumonia from pre-PCV13 period (2007–2009) to the post-PCV13 period (2011), although the difference was not statistically significant [26]. By comparison, we found a 13% and 16% decrease in the rate of index cases of pneumonia associated with hospitalization between 2008 and 2014 and between 2009 and 2014 respectively, but little change in the overall rate of pneumonia (+ 2% between 2008 and 2011 and − 5% between 2009 and 2011) or the rate of hospitalization for pneumonia (no change between 2008 and 2011 and − 4% between 2009 and 2011). Although pneumonia rates appear to have decreased somewhat since PCVs, especially PCV7, were introduced, these vaccines have had a greater impact on invasive pneumococcal disease [27], suggesting that pneumonia due to non-vaccine serotypes of *S. pneumoniae* or other causes remains a significant problem [15].

Limited data are available for comparison with our cost estimates. Park et al. estimated that the mean total pneumonia-related direct medical costs was $3376 in patients aged < 5 years, $4726 in patients aged ≥45 to ≤65 years, and $7206 in patients aged > 65 years [26]. However, their estimates reflect all pneumonia-related healthcare consumption within a year, not only the index episode as in our analysis.

This current study was strengthened by the large size and representativeness of the dataset, the use of real-world outpatient and inpatient settings, and the multiple years covered. Although we expect that our estimates are reliable, their accuracy could have been affected by misclassifications or measurement errors, for example, due to miscoding, misdiagnosis, or patients not using their insurance. Also, care should be taken when generalizing these results to other pneumonia patient populations with other types of health insurance coverage (e.g. Medicaid) or without insurance coverage.

## Conclusions

Our results indicate that pneumonia-associated healthcare utilization and costs remain substantial in the US, and they suggest that pneumonia due to non-vaccine serotypes of *S. pneumoniae* or other causes remains a significant problem. The results should be useful for estimating costs effectiveness of interventions and preventive measures, updating current therapeutic guidelines and policies, and justifying new drug and vaccine development. For example, data on age-specific pneumonia healthcare utilization can be applied to economic models to estimate whether vaccination programs targeted to young children or older adults are cost effective or cost saving [28–31].

Our results suggest that new or additional interventions beyond current pneumococcal vaccines are needed to further reduce the burden of pneumonia. Targeting vaccination and these further interventions especially to young children, elderly adults, and individuals with chronic medical conditions should substantially reduce the burden and costs of pneumonia in the US.

## Additional files

Additional file 1: **Table S1.** International Classification of Diseases, 9th revision, Clinical Modification (ICD-9-CM) codes used. (DOCX 13 kb)

Additional file 2: **Table S2.** Demographic characteristics of pneumonia patients by year. (DOCX 15 kb)

Additional file 3: **Table S3.** Frequency of index pneumonia visits per 1000 person-years by age group and year (DOCX 13 kb)

Additional file 4: **Table S4**. Proportion of pneumonia cases by setting, age group, and year (DOCX 15 kb)

## Abbreviations

CI: Confidence interval
ED: Emergency department
PCV13: 13-valent pneumococcal conjugate vaccine
PCV7: 7-valent pneumococcal conjugate vaccine
PY: Person-years
UC: Urgent care
