# Supplementary material for: Trends in healthcare utilization and costs associated with pneumonia in the United States during 2008–2014
Source: BMC Health Serv Res. 2018 Sep 14;18:715. doi: 10.1186/s12913-018-3529-4 (PMC6137867; doi:10.1186/s12913-018-3529-4)
Supplement: Supplementary file 1 — Table S1. International Classification of Diseases, 9th revision, Clinical Modification (ICD-9-CM) codes used. (DOCX 13 kb) [file 12913_2018_3529_MOESM1_ESM.docx]

**Table S1. International Classification of Diseases, 9th revision, Clinical Modification (ICD-9-CM) codes used**

| **Condition** | **Medical coding** | **ICD-9-CM code** |
| --- | --- | --- |
| Pneumonia | All pneumonia | 480-486 |
|  | Influenza with pneumonia | 487.0 |
| Meningitis | Meningitis due to other organisms | 321.xx |
|  | Tuberculous meningitis | 013.0 |
|  | Salmonella meningitis | 003.21 |
|  | Meningococcal meningitis | 036.0 |
|  | Meningococcal encephalitis | 036.1 |
|  | Meningitis due to enterovirus | 047.xx |
|  | Meningitis due to adenovirus | 049.1 |
|  | Herpes zoster with meningitis | 053.0 |
|  | Herpes simplex meningitis | 054.72 |
|  | Mumps meningitis | 072.1 |
|  | Acute syphilitic meningitis | 091.81 |
|  | Syphilitic meningitis | 094.2 |
|  | Gonococcal meningitis | 098.82 |
|  | Leptospiral meningitis (aseptic) | 100.81 |
|  | Candidal meningitis | 112.83 |
|  | Coccidioidal meningitis | 114.2 |
|  | Infection by Histoplasma capsulatum, meningitis | 115.01 |
|  | Infection by Histoplasma duboisii, meningitis | 115.11 |
|  | Histoplasmosis, unspecified, meningitis | 115.91 |
|  | Meningoencephalitis due to toxoplasmosis | 130.0 |
|  | Bacterial meningitis | 320.xx |
|  | Nonpyogenic meningitis | 322.0 |
|  | Meningitis, unspecified | 322.9 |
|  | Meningitis of unspecified cause | 322 |
| Septicemia | Salmonella septicemia | 003.1 |
|  | Septicemic plague | 020.2 |
|  | Anthrax septicemia | 022.3 |
|  | Disseminated due to other mycobacteria | 031.2 |
|  | Meningococcemia | 036.2 |
|  | Septicemia | 038.xx |
|  | Herpetic septicemia | 054.5 |
|  | Septic shock | 785.52 |
|  | Bacteremia | 790.7 |
|  | Sepsis | 995.91 |
|  | Severe sepsis | 995.92 |
| Empyema | Empyema | 510.xx |
