# Supplementary material for: Trends in healthcare utilization and costs associated with pneumonia in the United States during 2008–2014
Source: BMC Health Serv Res. 2018 Sep 14;18:715. doi: 10.1186/s12913-018-3529-4 (PMC6137867; doi:10.1186/s12913-018-3529-4)
Supplement: Supplementary file 2 — Table S2. Demographic characteristics of pneumonia patients by year. (DOCX 15 kb) [file 12913_2018_3529_MOESM2_ESM.docx]

**Table S2. Demographic characteristics of pneumonia patients by year**

|  |  |  |  |  |  |  |  |
| --- | --- | --- | --- | --- | --- | --- | --- |
| **Variable** | **2008** | **2009** | **2010** | **2011** | **2012** | **2013** | **2014** |
| **Pneumonia patients, n** | 486,442 | 618,116 | 611,186 | 758,196 | 751,418 | 581,317 | 579,383 |
| **Age (y)** |  |  |  |  |  |  |  |
| Mean (SD) | 41.5 (28.3) | 39.4 (28.4) | 41.8 (29.0) | 41.8 (29.3) | 40.5 (28.9) | 44.7 (29.1) | 43.5 (28.4) |
| Median | 46 | 42 | 46 | 46 | 43 | 50 | 48 |
| **Age range, n (%)** |  |  |  |  |  |  |  |
| < 1 y | 11,485 (2.4) | 13,405 (2.2) | 12,958 (2.1) | 14,650 (1.9) | 13,706 (1.8) | 9,873 (1.7) | 9,149 (1.6) |
| 1 y | 17,502 (3.6) | 21,448 (3.5) | 21,781 (3.6) | 24,531 (3.2) | 23,923 (3.2) | 17,365 (3.0) | 16,583 (2.9) |
| 2–4 y | 42,345 (8.7) | 55,947 (9.1) | 56,800 (9.3) | 67,586 (8.9) | 67,999 (9.0) | 47,525 (8.2) | 44,874 (7.7) |
| 5–17 y | 77,018 (15.8) | 119,234 (19.3) | 101,841 (16.7) | 136,775 (18.0) | 143,417 (19.1) | 87,542 (15.1) | 91,907 (15.9) |
| 18–49 y | 117,604 (24.2) | 148,653 (24.0) | 135,158 (22.1) | 164,332 (21.7) | 173,199 (23.0) | 124,349 (21.4) | 134,255 (23.2) |
| 50–64 y | 112,366 (23.1) | 131,416 (21.3) | 133,148 (21.8) | 158,501 (20.9) | 157,751 (21.0) | 128,960 (22.2) | 137,912 (23.8) |
| 65–74 y | 35,380 (7.3) | 43,561 (7.0) | 50,926 (8.3) | 64,542 (8.5) | 58,007 (7.7) | 58,449 (10.1) | 51,790 (8.9) |
| 75–84 y | 45,247 (9.3) | 51,375 (8.3) | 58,703 (9.6) | 72,805 (9.6) | 63,639 (8.5) | 60,146 (10.3) | 51,582 (8.9) |
| ≥ 85 y | 27,495 (5.7) | 33,077 (5.4) | 39,871 (6.5) | 54,474 (7.2) | 49,777 (6.6) | 47,108 (8.1) | 41,331 (7.1) |
| **Male, n (%)** | 237,577 (48.8) | 300,095 (48.5) | 297,041 (48.6) | 370,111 (48.8) | 367,006 (48.8) | 282,304 (48.6) | 280,429 (48.4) |
| **Insurance, n (%)** |  |  |  |  |  |  |  |
| Commercial ^a^ | 376,718 (77.4) | 488,159 (79.0) | 459,552 (75.2) | 563,590 (74.3) | 577,446 (76.8) | 413,048 (71.1) | 432,451 (74.6) |
| Medicare ^b^ | 109,724 (22.6) | 129,957 (21.0) | 151,634 (24.8) | 194,606 (25.7) | 173,972 (23.2) | 168,269 (28.9) | 146,932 (25.4) |
| **US geographic region, n (%)** |  |  |  |  |  |  |  |
| Northeast | 47,036 (9.7) | 81,102 (13.1) | 95,754 (15.7) | 157,767 (20.8) | 150,860 (20.1) | 117,906 (20.3) | 130,915 (22.6) |
| North Central | 145,137 (29.8) | 171,338 (27.7) | 167,520 (27.4) | 205,485 (27.1) | 182,067 (24.2) | 140,393 (24.2) | 139,629 (24.1) |
| South | 220,665 (45.4) | 274,831 (44.5) | 220,599 (36.1) | 236,864 (31.2) | 271,295 (36.1) | 184,477 (31.7) | 187,307 (32.3) |
| West | 71,515 (14.7) | 89,619 (14.5) | 121,671 (19.9) | 134,918 (17.8) | 128,924 (17.2) | 123,328 (21.2) | 107,470 (18.5) |
| Unknown | 2089 (0.4) | 1226 (0.2) | 5642 (0.9) | 23,162 (3.1) | 18,272 (2.4) | 15,213 (2.6) | 14,062 (2.4) |

Abbreviations: SD, standard deviation

^a^ Includes active employees and their dependents, early (non-Medicare) retirees and their dependents, and individuals covered under the Consolidated Omnibus Budget Reconciliation Act

^b^ Includes retirees (> 65 y), their dependents, and younger people with disabilities
