# Supplementary material for: Trends in healthcare utilization and costs associated with pneumonia in the United States during 2008–2014
Source: BMC Health Serv Res. 2018 Sep 14;18:715. doi: 10.1186/s12913-018-3529-4 (PMC6137867; doi:10.1186/s12913-018-3529-4)
Supplement: Supplementary file 3 — Table S3. Frequency of index pneumonia visits per 1000 person-years by age group and year (DOCX 13 kb) [file 12913_2018_3529_MOESM3_ESM.docx]

**Table S3. Frequency of index pneumonia visits per 1000 person-years by age group and year**

| **Age group** | **2008** | **2009** | **2010** | **2011** | **2012** | **2013** | **2014** |
| --- | --- | --- | --- | --- | --- | --- | --- |
| < 1 y | 34.4 | 34.7 | 31.0 | 30.8 | 28.9 | 25.7 | 22.5 |
| 1 y | 49.8 | 52.9 | 49.4 | 50.1 | 49.1 | 44.2 | 39.9 |
| 2–4 y | 38.2 | 43.2 | 39.5 | 41.6 | 42.6 | 37.3 | 33.9 |
| 5–17 y | 13.5 | 18.0 | 13.7 | 16.3 | 17.1 | 12.8 | 12.8 |
| 18–49 y | 8.2 | 8.9 | 7.2 | 7.6 | 7.8 | 6.8 | 6.8 |
| 50–64 y | 30.9 | 28.3 | 27.7 | 28.3 | 28.1 | 27.6 | 26.4 |
| 65–74 y | 15.2 | 14.8 | 13.3 | 13.8 | 13.6 | 13.3 | 13.1 |
| 75–84 y | 55.6 | 51.6 | 52.0 | 53.2 | 54.5 | 54.1 | 52.6 |
| ≥ 85 y | 95.6 | 84.3 | 91.4 | 95.5 | 98.5 | 98.6 | 95.7 |
| Overall | 15.4 | 16.6 | 14.6 | 15.7 | 15.5 | 14.3 | 13.5 |
