# Supplementary material for: Trends in healthcare utilization and costs associated with pneumonia in the United States during 2008–2014
Source: BMC Health Serv Res. 2018 Sep 14;18:715. doi: 10.1186/s12913-018-3529-4 (PMC6137867; doi:10.1186/s12913-018-3529-4)
Supplement: Supplementary file 4 — Table S4. Proportion of pneumonia cases by setting, age group, and year (DOCX 15 kb) [file 12913_2018_3529_MOESM4_ESM.docx]

**Table S4. Proportion of pneumonia cases by setting, age group, and year**

| **Age group** | **Setting** | **2008** | **2009** | **2010** | **2011** | **2012** | **2013** | **2014** |
| --- | --- | --- | --- | --- | --- | --- | --- | --- |
| **< 1 y** | Hospitalization | 13.8 | 12.6 | 13.1 | 11.4 | 11.1 | 10.4 | 10.8 |
|  | ED/UC visits | 23 | 23 | 24.1 | 26.6 | 26.7 | 26.9 | 26.6 |
|  | Outpatient visits | 63.2 | 64.4 | 62.8 | 62 | 62.2 | 62.7 | 62.6 |
|  |  |  |  |  |  |  |  |  |
| **1 y** | Hospitalization | 8.3 | 7.7 | 7.9 | 6.5 | 6.1 | 5.9 | 5.4 |
|  | ED/UC visits | 22.2 | 22.8 | 21.9 | 23.6 | 23.2 | 22.8 | 23.4 |
|  | Outpatient visits | 69.5 | 69.5 | 70.1 | 69.9 | 70.7 | 71.3 | 71.2 |
|  |  |  |  |  |  |  |  |  |
| **2–4 y** | Hospitalization | 5.4 | 5.1 | 5.1 | 4.1 | 3.9 | 3.6 | 3.7 |
|  | ED/UC visits | 15.3 | 16.1 | 15.1 | 16 | 15.4 | 15.9 | 16.6 |
|  | Outpatient visits | 79.4 | 78.7 | 79.7 | 79.8 | 80.7 | 80.5 | 79.8 |
|  |  |  |  |  |  |  |  |  |
| **5–17 y** | Hospitalization | 3.9 | 3.8 | 3.7 | 2.9 | 2.6 | 2.6 | 2.7 |
|  | ED/UC visits | 11 | 12.1 | 11.3 | 12.2 | 12.5 | 13 | 13.8 |
|  | Outpatient visits | 85.1 | 84.1 | 85 | 84.9 | 84.9 | 84.4 | 83.5 |
|  |  |  |  |  |  |  |  |  |
| **18–49 y** | Hospitalization | 8.4 | 8.8 | 9.3 | 8.5 | 7.7 | 8.1 | 8.4 |
|  | ED/UC visits | 18.3 | 18.9 | 19.1 | 21.6 | 23.7 | 25.7 | 27.9 |
|  | Outpatient visits | 73.4 | 72.3 | 71.6 | 70 | 68.5 | 66.2 | 63.7 |
|  |  |  |  |  |  |  |  |  |
| **50–64 y** | Hospitalization | 15.2 | 16 | 16.3 | 15.8 | 14.7 | 15.2 | 15.7 |
|  | ED/UC visits | 13.3 | 14.4 | 14.4 | 15.7 | 17.3 | 18.2 | 19.3 |
|  | Outpatient visits | 71.5 | 69.6 | 69.3 | 68.5 | 68 | 66.6 | 65 |
|  |  |  |  |  |  |  |  |  |
| **65–74 y** | Hospitalization | 28.2 | 28.3 | 28.2 | 27.3 | 26.3 | 26.2 | 27.4 |
|  | ED/UC visits | 12.8 | 13.8 | 13.8 | 15.3 | 15.9 | 15.2 | 15.3 |
|  | Outpatient visits | 59.0 | 57.9 | 58.0 | 57.4 | 57.7 | 58.6 | 57.3 |
|  |  |  |  |  |  |  |  |  |
| **75–84 y** | Hospitalization | 35.9 | 35.9 | 36.4 | 35.1 | 34.2 | 34.1 | 34.6 |
|  | ED/UC visits | 13.8 | 15.1 | 14.0 | 15.8 | 16.5 | 14.9 | 14.5 |
|  | Outpatient visits | 50.3 | 49.0 | 49.6 | 49.1 | 49.4 | 51.0 | 50.9 |
|  |  |  |  |  |  |  |  |  |
| **≥ 85 y** | Hospitalization | 40.2 | 39.9 | 39.7 | 38.1 | 37.8 | 37.0 | 37.4 |
|  | ED/UC visits | 13.7 | 15.3 | 14.6 | 15.6 | 16.2 | 14.6 | 13.8 |
|  | Outpatient visits | 46.1 | 44.8 | 45.7 | 46.2 | 46.0 | 48.4 | 48.8 |
|  |  |  |  |  |  |  |  |  |
| **Overall** | Hospitalization | 14.9 | 14.4 | 15.7 | 14.9 | 13.5 | 15.3 | 14.9 |
|  | ED/UC visits | 14.9 | 15.7 | 15.4 | 16.8 | 17.8 | 18.2 | 19.3 |
|  | Outpatient visits | 70.2 | 69.9 | 68.9 | 68.3 | 68.6 | 66.5 | 65.8 |

Abbreviations: ED, emergency department; UC, urgent care
